# Advances in the Study of the Tick Cattle Microbiota and the Influence on Vectorial Capacity

**DOI:** 10.3389/fvets.2021.710352

**Published:** 2021-08-17

**Authors:** Hugo Aguilar-Díaz, Rosa Estela Quiroz-Castañeda, Mayra Cobaxin-Cárdenas, Elizabeth Salinas-Estrella, Itzel Amaro-Estrada

**Affiliations:** ^1^Unidad de Artropodología del Centro Nacional de Investigación Disciplinaria en Salud Animal e Inocuidad, Instituto Nacional de Investigaciones Forestales, Agrícolas y Pecuarias, Morelos, Mexico; ^2^Unidad de Anaplasmosis del Centro Nacional de Investigación Disciplinaria en Salud Animal e Inocuidad, Instituto Nacional de Investigaciones Forestales, Agrícolas y Pecuarias, Morelos, Mexico

**Keywords:** bovine host, vector of transmission, metagenomics, microbiota, microbiome, vectorial capacity, ticks

## Abstract

The information from the tick cattle microbiota suggests that the microbial populations may modulate a successful infection process of the tick-borne pathogens. Therefore, there is a need to know the microbial population and their interactions. In this mini-review, we present several examples of how microbiota regulates the survival of pathogens inside the tick and contributes to fitness, adaptation, and tick immunity, among others. The communication between the tick microbiota and the host microbiota is vital to understanding the pathogen transmission process. As part of the tick microbiota, the pathogen interacts with different microbial populations, including the microorganisms of the host microbiota. These interactions comprise a microsystem that regulates the vectorial capacity involved in tick-borne diseases. The knowledge we have about the vectorial capacity contributes to a better understanding of tick-borne pathogens. Additionally, using approaches based on multi-omics strategies applied to studying the microbiota and its microbiome allows the development of strategies to control ticks. The results derived from those studies reveal the dynamics of the microbiota and potential targets for anti-tick vaccine development. In this context, the anti-microbiota vaccines have emerged as an alternative with a good prognosis. Some strategies developed to control other arthropods vectors, such as paratransgenesis, could control ticks and tick-borne diseases.

## Introduction

**Figure 1:**
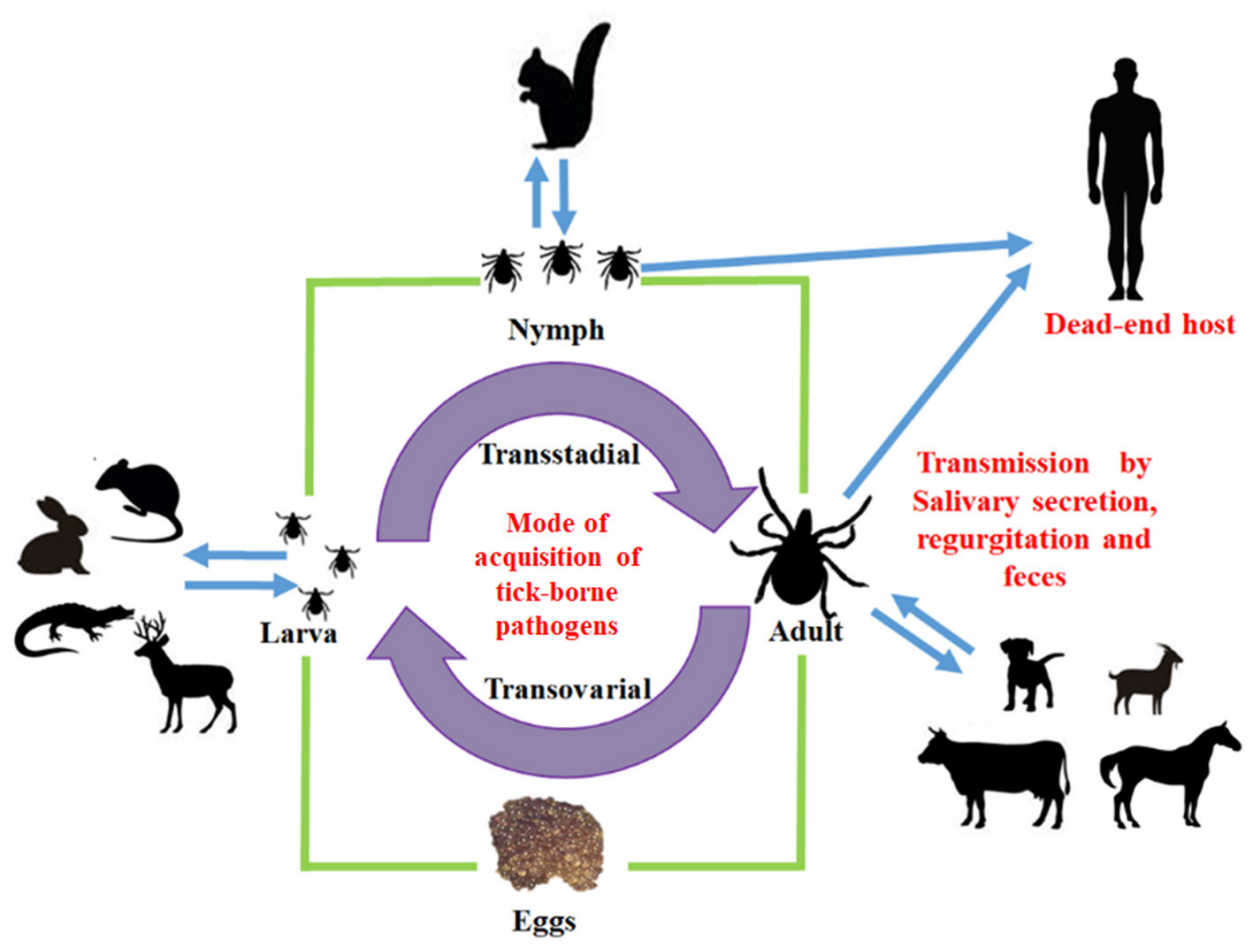
Mode of acquisition of tick-borne pathogens. The different stages of tick (larva, nymph, and adult) may transmit bacteria to intermediate or final host.

Infectious diseases have been one of the main restrictions worldwide for animal production improvement, with significant economic losses. The significant risk that endangers animal health is the arthropod vector and the pathogens they transmit and those that have been controlled or eradicated, and after a while, they reemerge. Ticks are hematophagous ectoparasites, the main biological vectors of numerous infectious diseases (tick-borne diseases) (1–3) (Figure 1).

Although the study of the tick microbiota and the biological processes in which it participates are still in progress to date, there is not enough information about its role in the vectorial capacity, infection, and pathogen transmission. This study opens up a research field to study and elucidate new targets for developing drugs/vaccines to prevent diseases that affect animals, including humans. The development of anti-tick vaccines based on the microbiota represents a promising approach to control tick infestations. In this regard, the study of the tick microbiota contributes to elucidate the interactions that may be influencing vital processes and then avoiding the transmission of pathogens (4, 5). However, there is much work to obtain an effective drug or vaccine to control tick-borne diseases and the pathogens that cause them.

## A Brief Overview of Tick-Borne Diseases In Cattle

During the last years, the number of tick-borne diseases caused by bacteria *Anaplasma, Ehrlichia*, and *Coxiella* has increased, affecting livestock productivity (6). *Anaplasma marginale* causes bovine anaplasmosis resulting in economic losses to the cattle industry due to a significant reduction of beef and dairy production (7). More recently, some coinfections with piroplasmas and other *Anaplasmataceae* reported in cattle and buffaloes show the circulation of diverse genotypes of *A. marginale* worldwide (8).

So far, there exist more than 20 genomes and draft genomes of *A. marginale*, whose content could contribute to the identification and participation of the genes involved in the microbiota–vector–host interaction (9–11).

*Ehrlichia ruminantium* is transmitted by *Amblyomma* and causes *Heartwater*, the most important livestock disease in Africa and the Caribbean, while the emerging *Ehrlichia minasensis* also infects cattle (12–14). As an example of the intrinsic interaction between pathogen and the host cell, molecular studies reveal that the genome of *Ehrlichia* sp. has lost genes associated with metabolism whose activities are covered by the host cell (15). In the genomic context, only a few genomes are available for *Ehrlichia* species (16, 17).

In European livestock, coxiellosis, caused by *Coxiella burnetii* and transmitted by *Hyalomma* spp. and *Rhipicephalus* spp. has a significant prevalence in the Mediterranean countries (18).

At the moment, there exist more than 75 *Coxiella* genomes reported that contain a large number of genes participating in adhesion, invasion, intracellular trafficking, host-cell modulation, and detoxification (19).

In Africa, Ben Said et al. (20) interestingly identified the spirochaetes, *Borrelia burgdorferi*, in goats, sheep, camels, and cattle, transmitted by *Rhipicephalus* and *Hyalomma*. The recent molecular detection of *Rickettsia* spp. and *C. burnetii* in cattle and water buffalo in Luzon Island of the Philippines reveals the potential zoonotic transmission mediated by *Rhipicephalus microplus* (21).

Hemoplasmosis is not strictly considered a tick-borne disease; however, *Mycoplasma wenyonii* and *Candidatus* Mycoplasma haemobos reported in cattle are probably transmitted by ticks (22–25).

## Cattle Tick Microbiota Interactions and Vectorial Capacity

The microbial communities that comprise the tick microbiota include pathogens, symbionts, and commensals acting as a dynamic and integrative microecosystem, changing in time and scale, that interact into a macrosystem that includes the host (26, 27). Additionally, external factors modulate the diversity of the microbiota: temperature, humidity, geographic location, sex and species, blood intake from the vertebrate host, or even the physical location inside the organs of the tick (gut, ovaries, salivary glands) (28, 29).

Interestingly, this microbiota also contributes to fitness, nutritional adaptation, development, and reproduction and is also involved in establishing pathogens inside the tick (30, 31).

Currently, there exists a close relationship between pathogens and the tick microbiota, which affects their vectorial capacity. Also, colonization, replication, or maturation of an infective form of a pathogen depends mainly on the microbiota composition.

During the blood intake, the hematophagous vector can acquire pathogens from an infected host that transmit to a new host. This is known as vector competence, the ability of a vector to maintain the pathogen development until its transmission to a new host. Vector competence is a component of vectorial capacity that comprises the interactions between vector–pathogen and vector–host, influenced by behavioral and environmental factors such as vector density, longevity, host preference, and feeding habits (32).

**Figure 2:**
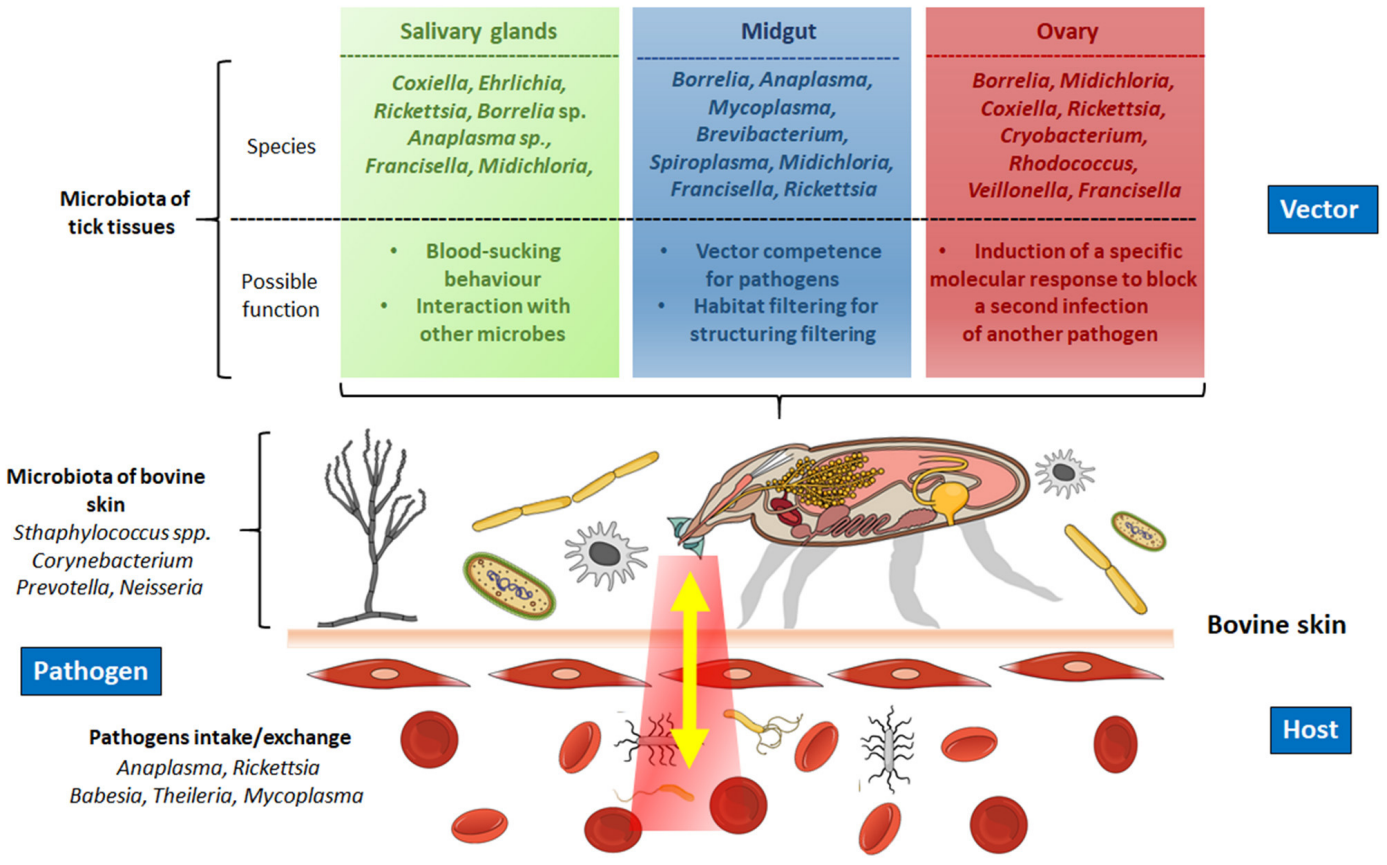
Modulation of the interaction microbiota–pathogen–tick–host during blood intake. Upper panel, the microbiota of the tick interacts with the pathogens ingested during blood intake in different organs of the tick (salivary glands, gut, ovary). The microbiota of these organs promotes the establishment and reproduction of the pathogens and, in some cases, in the vectorial capacity to transmit the definitive host. Lower panel, interaction of the microbiota of the bovine blood with the microbiota of the tick. The microbiota of the bovine's skin also interacts with the tick. Created in the Mind the Graph platform.

In tick cattle, the study of interaction microbiota–vector is still scarce. However, the results reported in other species could be applied as alternatives to identify new targets to control ticks. In addition, after blood intake, some complex interactions are carried out by the triad microbiota–vector–host involved in the pathogen transmission from one host to another (Figure 2). In this regard, the first interaction occurs between the tick and the microbiota of the bovine skin, in which several families of bacteria are present, including *Corynebacteriaceae* and *Staphylococcaceae* (teat skin), and *Firmicutes, Spirochaetae, Bacteroidetes*, and *Actinobacteria* (interdigital skin) (33, 34) (Figure 2). The microorganisms penetrate from the surface of the skin host deeper into the dermis and might be inducing local immunomodulation (35). For instance, the bacteria *Prevotella* sp. and *Neisseria* sp., usually found in the skin and mucosal surface of the host, respectively, have been identified in the midgut of *I. ricinus* blood-fed females, regardless of the time point of the blood feeding course (36, 37). In contrast, Miranda-Miranda et al. (38, 39) found that bacteria *Staphylococcus saprophyticus* and *Staphylococcus xylosus*, located in the skin of bovine, can produce a lethal infection in fully engorged female ticks that lose the ability to oviposit and lead them eventually to death. These findings show that the composition of the microbiota does not always favor tick infestations and pathogen transmission.

A second interaction occurs when the microbiota of the salivary glands is in contact with the pathogen that enters the tick. Here, the microbiota has an essential interaction with the pathogens, as in *Amblyomma americanum*, where the *Coxiella-*related symbionts in the salivary glands impair the transmission of *Ehrlichia chaffeensis* (40). These studies illustrate the pathogen interactions occurring in the salivary glands and highlight the role of the tick microbiota that regulates pathogen growth. Alternatively, during the pathogen inoculation, the microbiota of the saliva interacts with the host's components regulating the pathogen passage from the salivary glands to a new bite site.

The tick gut microbiota possesses different microorganisms that participate in metabolic and digestive processes. In this tissue, the blood and the pathogens ingested interact and alter the microbiota, and a third interaction occurs. In such a way, the altered microbiota, which usually provides cofactors, vitamins B, and folate (e.g., *Coxiella, Francisella, Rickettsia*, respectively), may affect the tick's development, as occurs in *Ornithodoros moubata*, where antibiotic-based elimination of *Francisella* endosymbionts compromise the nutritional status of vitamin B, which results in some anomalies in tick development and hampers nymph growth and molting to adults (41). Another role of the microbiota is to regulate the pathogen colonization of the tick gut. For example, in *Ixodes scapularis* a gut microbiota composed of a high abundance of *Rickettsia, Thioclava*, and *Delftia*, and a low abundance of *Aquabacterium, Brevibacterium*, and *Novosphingobium* may influence *B. burgdorferi* colonization negatively (42).

Once established, the pathogens cross the peritrophic membrane and the gut barrier to disseminating to other tissues through the hemolymph. Here, the presence of hemocytes and effector molecules of the tick immune response represents a hostile environment for the pathogen; however, the information about the role of the microbiota of the hemolymph and its interaction is scarce except for the finding of *Staphylococcus aureus* in *Rhipicephalus decoloratus* and *Rhipicephalus geigy*, which were isolated from the adult females hemolymph (43).

Finally, the pathogen could likely take two routes. The first, pathogens redirect to the salivary glands and transmit to a new host, and the second, pathogens transmit by transovarial transmission. In both cases, the pathogen would be interacting with the microbiota of the tissue.

Although the information about the effect of tick-borne pathogens and microbiota is scarce, the report of co-occurences in epidemiological studies has suggested the impact of pathogens on the tick and vice versa (27). For example, the microbial community in the tick gut is related to the virulence and proliferation of *Anaplasma* sp. and *Babesia* sp. (27). Adegoke et al. (44) reported that microbial diversity and composition of *R. microplus* decreases when infected with *Theileria* sp.; this phenomenon is called pathogen-induced dysbiosis. A similar effect occurs in *D. andersoni* where endosymbiont *Francisella* sp. increases the successful pathogenic colonization of *Francisella novicida* and *A. marginale* (45, 46).

To address the potential role of the *I. scapularis* gut bacteria in modulating *B. burgdorferi*, (42) compared dysbiosed larvae and larvae fed on gentamicin-treated mice. They found that both types of larvae significantly increased engorgement weights and decreased *B. burgdorferi* colonization, suggesting that tick-altered gut microbiota impairs pathogen colonization.

On the other hand, the induction of *I. scapularis* antifreeze glycoprotein (IAFGP) occurs in the presence of *A. phagocytophilum*, which sequesters IAFGP to alter its accumulation in the tick midgut to inhibit the development of biofilms, thus allowing the colonization and establishment of the vector (47, 48).

Understanding how microbial diversity of ticks changes in the presence of pathogens could help propose better strategies to prevent colonization and establishment in the vector (49). Also, the interaction of the tick microbiota with the host microbiota affects vector competence; therefore, the elucidation of the mechanisms involved in these interactions allows the identification of molecular drivers for tick-borne disease (50).

## Metagenomic Approaches to Study Pathogens Transmitted by Ticks

So far, we have known that ticks have a great capacity to transmit various pathogens that cause different diseases in cattle. However, the function of the microbiota within the tick life cycle is still unclear due to the significant limitation of their isolation, cultivation, and propagation in culture media and the difficulty of infesting animals with ticks under controlled experimental conditions (30, 51). Because of these drawbacks, the use of next-generation sequencing, molecular techniques, and or multi-omics technologies represents valuable tools in the study of the tick microbiota and its microbiome.

In this regard, the use of 16S rRNA gene-targeted metagenomics provides new insights into tick-borne pathogens (52). The use of this technique has allowed the identification of bacteria in ticks such as *R. microplus, Hyalomma anatolicum*, and *Haemaphysalis montgomery* from cows, where *Ralstonia, Staphylococcus*, and *Francisella* were some genera detected (53). Metagenomics studies show that the microbiota of *R. microplus* captured in a rural area in Peru has 147 bacterial genera (54). In contrast, the sequencing of internal tissue and salivary glands from unfed larvae and female ticks of *I. ricinus* revealed commensal bacteria, endosymbionts, and several pathogenic microorganisms (54, 55). Also, metagenomic analysis of *Ixodes persulcatus* and *Dermacentor nuttalli* revealed 10 predominant genera of cattle pathogens and also coinfections (56).

A deeper metagenomic study elucidated the taxonomic and functional profiles of the microbiome of female and male ticks, *Ixodes ovatus, I. persulcatus*, and *Amblyomma variegatum* (57). The results of this study showed significant differences in the abundance of genes involved in metabolic pathways between female and male ticks of the same species, suggesting their different lifestyles exert and sex-specific evolutionary pressure independently of the phenomes and microbiomes of the tick gut microbiota.

Complementary information to metagenomics studies in microbiota derives from other multi-omics strategies such as metabolomics, transcriptomics, and proteomics that contribute to advancing knowledge of pathogens and their interactions with the host and vector (58), as in the case of the studies that show diverse components of tick saliva are capable of modulating host immune response through the binding to cell receptors and regulating the secretion of cytokines, chemokines, and interleukins (35). However, we propose that tick microbiota requires an interdisciplinary approach, where the metagenomics study combined with other multi-omics tools complements the results to give a complete vision of the microbiota–vector–host interactions.

## Paratransgenesis as a Strategy to Control Ticks

Based on the progress of metagenomics study applied to identify microbiota in other arthropods vectors, such as *Aedes, Culex*, and *Anopheles*, strategies such as paratransgenesis could control tick infestations. This strategy is an innovative method with a good prognosis in the vectors control. This strategy requires knowledge of the tick microbiota. After the symbiont microorganisms' identification, they are isolated and genetically transformed to generate bacteria capable of expressing the specific inhibitory molecules and then reintroduced to obstruct vital biological processes of the vector. In this regard, some reports suggest that the interference of crucial processes such as ovogenesis and vitellogenesis decreases the fecundity, development, and hatching rates, which results in the inhibition of the vector population growth (59). Similarly, another target is the transformation of bacteria that interfere with the digestive capacity, leading to reduced vector reproduction and preventing pathogen dissemination (59). Adopting strategies based on metagenomic studies allows identifying symbiont bacteria in the tick microbiota that can be genetically modified and cultivated with higher efficiency, which overcomes the need to produce genetically modified vectors (60, 61).

## Microbial Targets: Anti-Tick Vaccines

In response to the presence of acaricide-resistant populations, several vaccine proposals were developed in the last years, including the use of antigens such as Gavac, a vaccine based on protein Bm86 (62). The formulation of cocktail anti-tick vaccines has been reported using combinations of antigens, like Bm91 (*R. microplus*); subolesin 4E6 (*I. scapularis*); serpins rRAS-2 and rRAS-4 (*R. appendiculatus*); and glutathione S-transferase rGST-Av (*A. variegatum*), among others (63).

Currently, the use of experimental anti-tick vaccines targeting the microbiota reveals that modifications in the microbial populations of the gut could alter essential processes in a tick. For instance, the use of this experimental anti-tick vaccine developed after a functional metagenomic analysis shows that immunization of α-1,3-galactosyltransferase-deficient mice with *Escherichia coli* BL21 induces the production of anti-*E. coli* and anti-α-Gal IgM and IgG associated with mortality of *I. ricinus* nymphs during feeding, which concurred with the abundance of α-1,3-galactosyltransferase genes and possibly α-Gal identified by tick microbiome metagenomics analysis (5).

Finally, the search for new vaccine targets should encompass those proteins of the tick immune response involved in the tolerance of microbial populations (tick microbiota), like different proteins identified by immunoinformatic analysis (64).

## Conclusions

Here, we show a general view of different approaches for studying the tick microbiota and other arthropod vectors, intending to integrate the current knowledge and present new alternatives for tick control. From our perspective, in this mini-review, we include recent results that show that the more knowledge and manipulation we have of the microbiota, we get closer to new vaccine development.

The study of the tick microbiota based on metagenomics approaches allows identifying microorganisms and can elucidate the genes that shape the microbiome.

Although the microbiota participates in biological processes like adaptation, development, reproduction, defense against environmental stress, and immunity, information about the interactions and mechanisms involved in vectorial capacity is still scarce and, at the same time, is a field with potential for the identification of vaccine targets.

Considering that many biological interactions function as holobionts (host organism and its associated microbial community) and that, in turn, holobionts make up a more extensive consortium, it is impossible to think that the tick microbiota and tick-borne pathogens are isolated processes.

Undoubtedly, deciphering the interaction of the tick and host–microbiota and how they communicate will provide invaluable information to develop novel strategies for controlling ticks and vector-borne pathogens, like those anti-microbiota and anti-tick vaccine candidates that could benefit animal health and provide acaricide-free environments.

## Author Contributions

REQ-C and HA-D: conceptualization. REQ-C, HA-D, ES-E, MC-C, and IA-E: investigation and original draft preparation and writing and draft preparation. REQ-C and HA-D: review and editing. All the authors have read and agreed to the published version of the manuscript.

## Conflict of Interest

The authors declare that the research was conducted in the absence of any commercial or financial relationships that could be construed as a potential conflict of interest.

## Publisher's Note

All claims expressed in this article are solely those of the authors and do not necessarily represent those of their affiliated organizations, or those of the publisher, the editors and the reviewers. Any product that may be evaluated in this article, or claim that may be made by its manufacturer, is not guaranteed or endorsed by the publisher.
